# Supplementary material for: On local intrinsic dimensionality of deformation in complex materials
Source: Sci Rep. 2021 May 13;11:10216. doi: 10.1038/s41598-021-89328-8 (PMC8119735; doi:10.1038/s41598-021-89328-8)
Supplement: Supplementary file 1 — Supplementary material 1 (pdf 3759 KB) [file 41598_2021_89328_MOESM1_ESM.pdf]

Supplementary Information

On local intrinsic dimensionality of deformation in complex materials

Shuo Zhou, Antoinette Tordesillas, Mehdi Pouragha, James Bailey, and Howard Bondell

Learning the cutoff value

Given the s-LID of particles in the system, a cutoff value, α^*, is used to classify particles into two categories: a collection of highly abnormal particles with s-LID larger than α^*, and the rest. The idea of using a threshold to differentiate particles is inspired by force chain identification algorithms[1–3]. Findings suggest that average force can be used as a cutoff value to identify a subset of contacts/particles with higher than average force, which are usually compactly connected in the physical space, forming the so-called force chains. Similarly, we categorize particles in the system based on their s-LID values compared to a learned cutoff value, in order to identify localization structures.

To learn the cutoff value, we build on the recent advances in explosive percolation[4,5]. A critical transition point can be found in the growth process of the complex networks that are containing sub-groups, where densely connected communities are bridging via nodes of relatively sparse connectivity. Achlioptas et al.[4] employed an edge selection procedure that randomly adds new edges to a network with the objective to minimize the size of the largest connected component (SLC) in the network. This procedure promotes the formation of multiple coexisting similar-sized connected components in the beginning, resulting a smooth increase of SLC with the addition of new edges. While in the long-run, abrupt explosive percolation can be found in SLC as existing components are connected by new edges. Similarly, Singh and Tordesillas[5] developed a new process by connecting pairs of particles in the granular material whose kinematic distances are under a given radius. By tracking the change of SLC with the increase of radius, sub-groups of grains moving in near-rigid body motion[6,7] were found at the critical transition radius. Motivated by these work, we propose to learn the critical s-LID cutoff, α^*, by designing a shrinking process in the contact network, in order to find a strong sub-network that is constituted of particles with relatively higher s-LID. Specifically, let $\alpha = \beta \cdot \gamma$ and γ be the average s-LID among all particles, we eliminate particles (and the associated edges) with s-LID $\leq \alpha$ from the contact network, and track the change of SLC in the remaining network while progressively increasing β. The β^* that is corresponding to the steepest drop in SLC indicates the sudden split of the network into multiple sub-groups, thus, $\alpha^* = \beta^* \cdot \gamma$ gives the best threshold value to divide the system into two distinct sub-groups.

The results of changes in SLC with the increase of β for each sample can be found in Figure S1. According to our analysis, in most of the cases, the SLC starts to decrease from $\beta = 0.5$ until $\beta = 1.5$, and the most dramatic drop in SLC can be found near $\beta = 1$, suggesting that similar to force chain identification, the means s-LID among all particles can be the optimal cutoff value to split the particles in the system into two groups.

References

1. Radjai, F., Wolf, D. E., Jean, M. & Moreau, J.-J. Bimodal character of stress transmission in granular packings. *Phys. Rev. Lett.* **80**, 61 (1998).
2. Peters, J., Muthuswamy, M., Wibowo, J. & Tordesillas, A. Characterization of force chains in granular material. *Phys. Rev. E* **72**, 041307 (2005).
3. Walker, D. M. *et al.* Percolating contact subnetworks on the edge of isostaticity. *Granul. Matter* **13**, 233–240 (2011).
4. Achlioptas, D., D'Souza, R. M. & Spencer, J. Explosive percolation in random networks. *Science* **323**, 1453–1455 (2009).
5. Singh, K. & Tordesillas, A. Spatiotemporal Evolution of a Landslide: A Transition to Explosive Percolation. *Entropy* **22**, 67 (2020).
6. Tordesillas, A., Walker, D. M., Rechenmacher, A. L. & Abedi, S. Discovering community structures and dynamical networks from grain-scale kinematics of shear bands in sand. In *IWBDG*, 67–73 (2011).
7. Tordesillas, A., Walker, D. M., Andò, E. & Viggiani, G. Revisiting localized deformation in sand with complex systems. *Proc. Royal Soc. A: Math. Phys. Eng. Sci.* **469**, 20120606 (2013).

Supplementary Figures

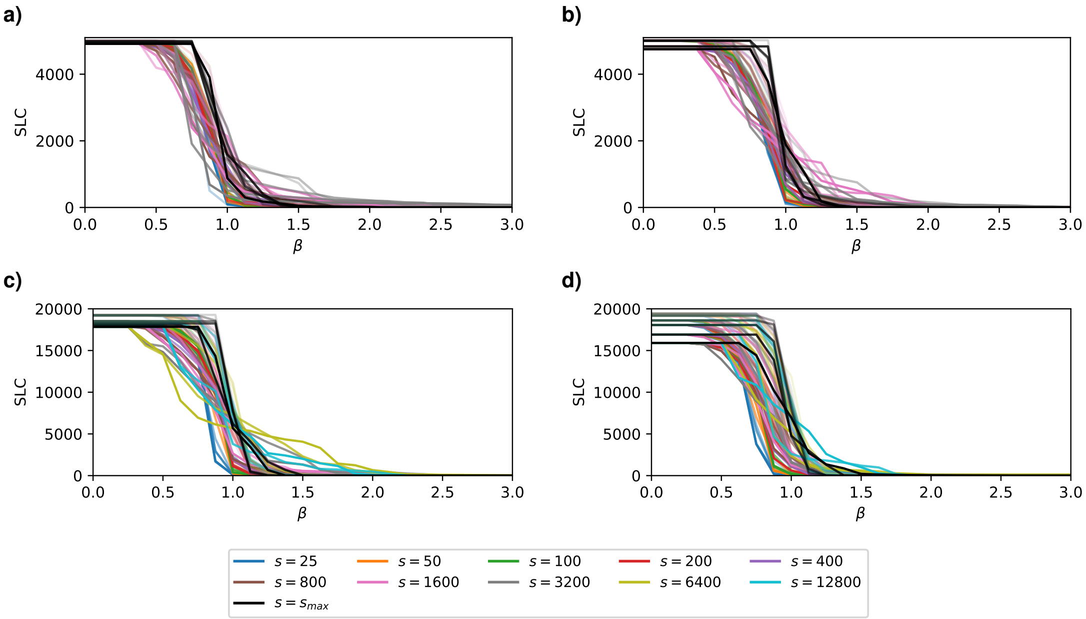

Figure S1. Change of SLC with the increase of β in different systems **(a)** 5K, **(b)** 5K-SR, **(c)** 20K, and **(d)** 20K-NR. The gradients in the same color indicate the results for the same neighborhood size, but at different strain stages of the loading history.

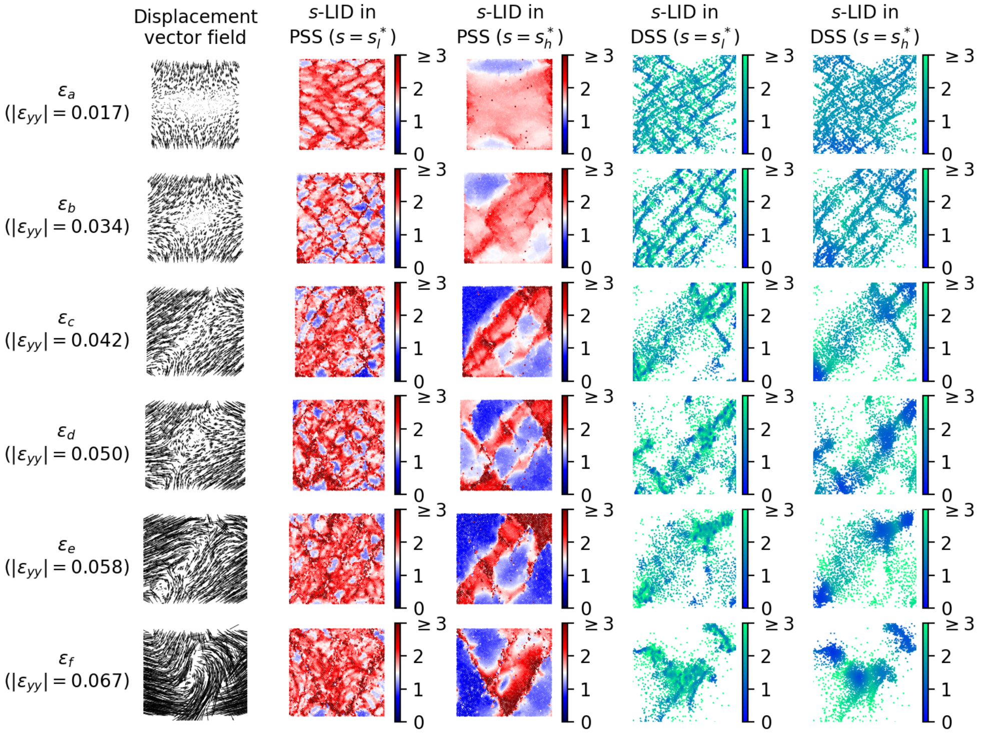

Figure S2. Visualization of displacement vector field, *s*-LID values of particles in DSS and PSS at different stages of the loading history for sample 5K-SR.

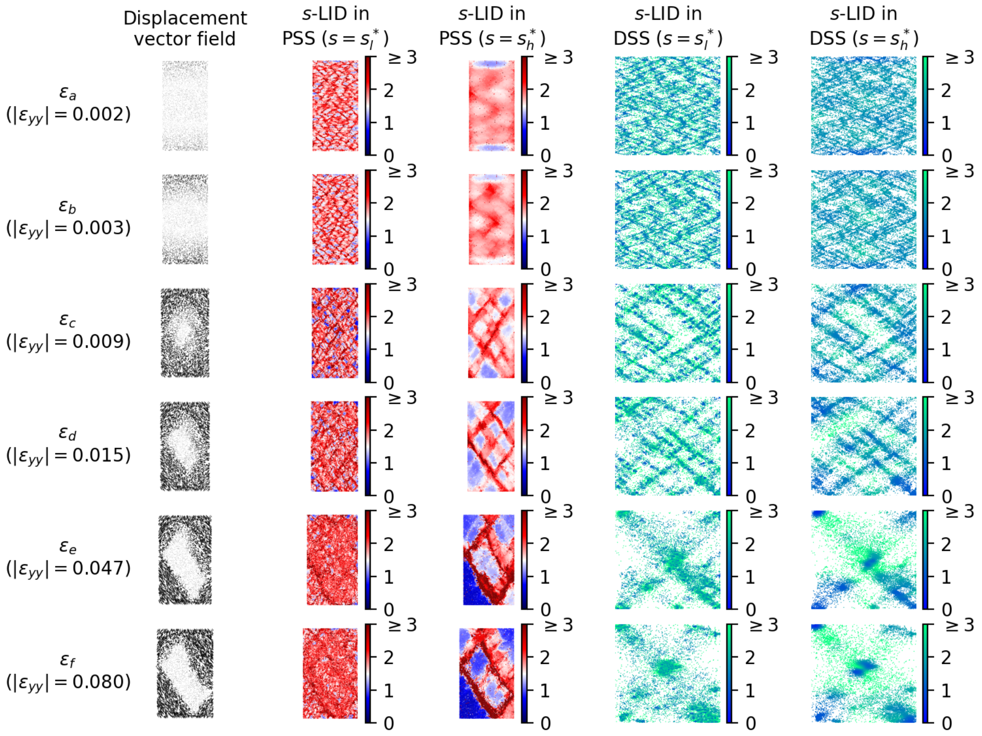

Figure S3. Visualization of displacement vector field, s-LID values of particles in DSS and PSS at different stages of the loading history for sample 20K.

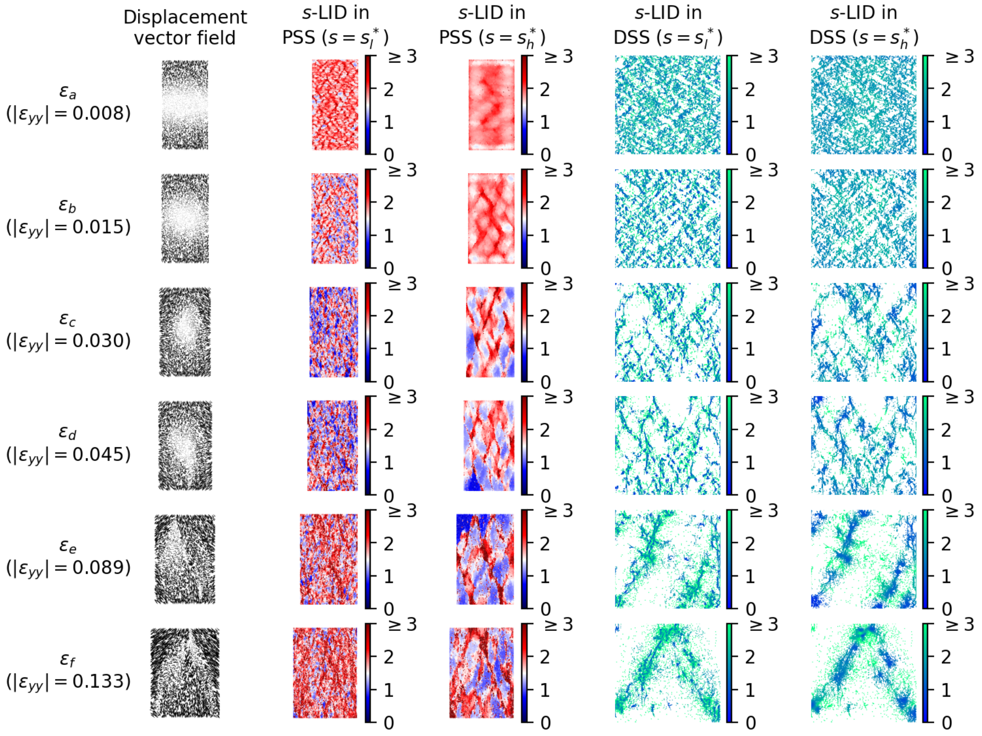

Figure S4. Visualization of displacement vector field, s-LID values of particles in DSS and PSS at different stages of the loading history for sample 20K-NR.

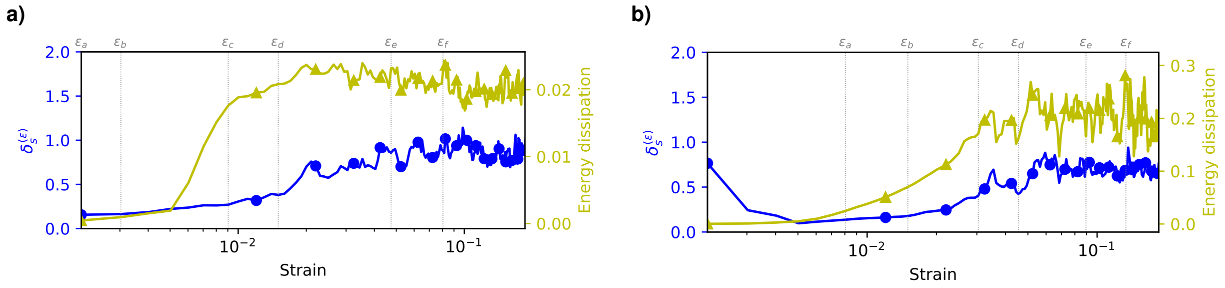

Figure S5. The evolution in the strength of shearband pattern measured in contrast and the energy dissipation in systems **(a)** 20K, and **(b)** 20K-NR. Note strains are shown in log scale for ease of presentation. Bursts to a peak in dissipation can be seen to correlate well with the contrast in shearband pattern.
